# Use of Topical Insulin in the Wound Healing Process After Ultrasonography-Guided Modified Deroofing Surgery in a Hidradenitis Suppurativa Patient: A Case Report

**DOI:** 10.7759/cureus.47836

**Published:** 2023-10-27

**Authors:** Yusuf Can Edek, Esra Adışen

**Affiliations:** 1 Dermatology, Gazi University, Ankara, TUR

**Keywords:** ultrasonography, surgery, insulin, hidradenitis suppurativa, deroofing

## Abstract

Hidradenitis suppurativa (HS) is an inflammatory disease causing nodules, abscesses, sinus tracts, and scars in fold areas. It significantly impacts patients' quality of life. Surgical treatments are becoming popular in dermatology, with deroofing being a common procedure. However, recurrence rates can be high due to not removing fibrotic areas and scar tissue entirely. Recent efforts have focused on removing these tissues to achieve lower recurrence rates. This case report describes a male patient with HS who underwent a newly defined ultrasonography-guided modified deroofing surgery for HS. The wound-healing process was then accelerated with the application of topical insulin. In this case, we would like to highlight the significance of using ultrasonography before HS surgery, confirm the importance of modified deroofing surgery, and emphasize that insulin can be used as an effective supplementary treatment for ulcer management.

## Introduction

Hidradenitis suppurativa (HS) is an inflammatory disease characterized by nodules, abscesses, sinus tracts, and scars located in fold areas such as the axilla, inguinal region, sub-mammary folds, and perianal region. The disease significantly affects patients' quality of life with accompanying pain and discharge. Follicular hyperkeratosis is a known trigger for the disease, even if the etiopathogenesis of the disease is not fully understood. HS treatment can be challenging, and combining various treatment methods can help increase treatment success. Surgical treatment options are now coming to the fore to control the disease, and their applications in dermatology practice are becoming popular [1,2]. In this case report, we present a male patient with HS who underwent a novel ultrasonography-guided modified deroofing surgery. The wound-healing process was then expedited using topical insulin, a popular adjunct therapy for ulcer management. Our case report defines the features of the modified deroofing surgery technique. It emphasizes the accelerating effect of the topical insulin application on every kind of wound healing, including surgery procedures.

## Case presentation

**Figure 1:**
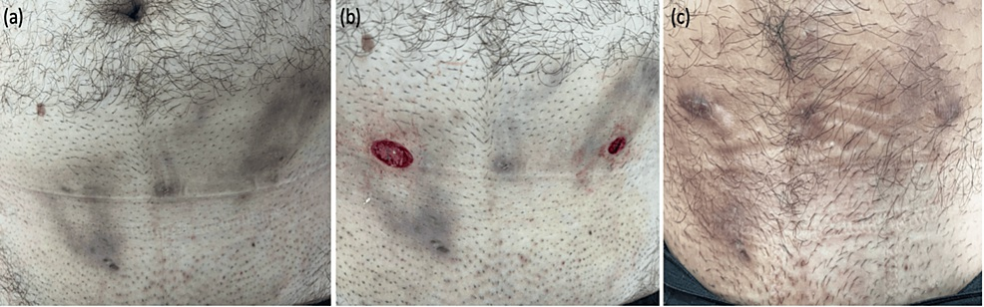
(a) Nodules and fistula tracts on the abdomen and inguinal area, (b) Area after excision, (c) Complete healing of the excised area by the 2.5th week post-surgery.

**Figure 2:**
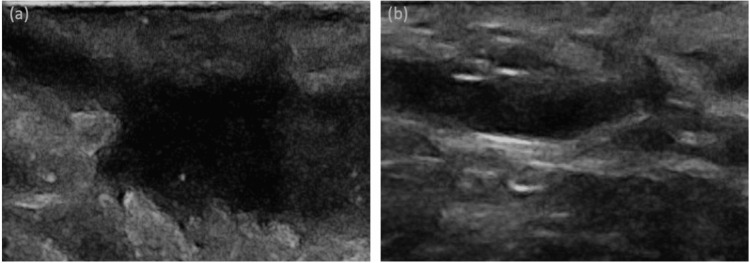
(a) and (b) Anechoic fluid collections and sinus tracts observed in the ultrasound scan of the lesions.

A 30-year-old male patient diagnosed with HS presented to our dermatology outpatient clinic. He had no known systemic diseases. For two years, he experienced recurrent pain, discharge, and swelling in the abdomen and inguinal regions. During this time, he had been using systemic antibiotics, specifically doxycycline, and a rifampicin-clindamycin combination. A dermatological examination of the patient showed nodules and fistula tracts located on the abdomen and inguinal area (Figure 1a). Anechoic fluid collections, pseudocysts, and sinus tracts were seen in the ultrasound scan of the lesions (Figure 2). The patient's Hurley stage was 2, the International Hidradenitis Suppurativa Severity Score System (IHS-4) score was 11, and the Dermatology Life Quality Index (DLQI) was 9. Since the lesions in the abdomen affected the patient's quality of life, we decided to perform modified deroofing surgery on the lesions. Before surgery, the borders of the excision area were determined with cutaneous ultrasonography. The procedure was performed under sterile conditions using local anesthesia. Lesions were excised down to the level of the subcutaneous fat tissue using an electrocautery device operating at 3.8 MHz in both cutting and coagulation modes. Finally, after the coagulation, we applied insulin treatment (Insulin aspart 100IU/ml® - 0.5 cc per ulcer, weekly) to the ulcerated area by dropping it to the ulcer borders with an insulin syringe and then covering it with a tight bandage. The ulcers quickly healed and re-epithelialized, and in 2.5 weeks, complete healing was achieved (Figure 1).

## Discussion

Deroofing is one of the most commonly performed procedures in dermatology for HS surgery. Deroofing is a surgical procedure in which the roof of the sinus tract is removed and left for secondary healing [2-4]. Following deroofing surgery, increased recurrence rates can be observed because of not removing fibrotic areas and scar tissue entirely. Thus, recent efforts have been made to remove these tissues to achieve lower recurrence rates. This is defined by Zaayman M et al. and called MOdified DEroofing With Scar Excision (MODES Procedure) [5]. In their study, Dahmen RA et al. confirmed lower recurrence rates of modified deroofing (14%) compared to standard deroofing (27%). In addition to recurrence rates similar to wide excision, the study also demonstrated a healing process akin to the standard deroofing procedure [6,7]. In recent years, cutaneous ultrasonography has been used to determine the boundaries of the surgery area before the procedure to determine inflammatory collections and tunnels that are difficult to detect solely by clinical examination and palpation [8]. In our case, we choose to perform the modified deroofing technique under the guidance of cutaneous ultrasonography, which allows a low recurrence rate. Applications designed to speed up the wound healing process are required to achieve quick and effective wound healing since the excised area in the modified deroofing procedure is larger than in standard deroofing. The topical use of insulin in wound management has become increasingly popular in recent years. Data on the use of topical insulin in several conditions, including leg ulcers, pressure ulcers, and surgical wounds, can be found in the literature. Insulin exerts its effects by triggering the migration of keratinocytes, fibroblasts, and endothelial cells to the wound area and playing a role in angiogenesis and granulation tissue formation [9,10]. Due to the beneficial effects of insulin on wound healing, we applied it to post-surgical wounds. As a result, wound healing was swift and free of complications. Dahmen RA et al. observed complete epithelialization in an average of 5.2 weeks (2.3-8.0) following modified deroofing, while we detected it in the 2.5th week in our case [6]. This accelerated healing is essential to demonstrate insulin's effect on wound healing.

## Conclusions

In this case report, we detail the cutaneous ultrasonography-guided modified deroofing technique. We emphasize the value of cutaneous ultrasonography prior to HS surgery, confirm the necessity of modified deroofing surgery, and state that insulin can be applied as an effective add-on therapy for ulcers.
